# Supplementary material for: The Resource Identification Initiative: A cultural shift in publishing
Source: F1000Res. 2015 Nov 19;4:134. Originally published 2015 May 29. [Version 2] doi: 10.12688/f1000research.6555.2 (PMC4648211; doi:10.12688/f1000research.6555.2)
Supplement: Peer review history

# Peer review: Referee response for version 1

**DOI:** 10.5256/f1000research.7039.r8811

**Published:** 2015-06-18

**Authors:** William Gunn

**Affiliations:** 1 Mendeley and the Reproducibility Initiative, Mountain View, CA, USA

The aim of this study was to determine the feasibility and utility of asking authors to include RRIDs when submitting a manuscript. The process for making these determinations was well-considered and the experience and professional relationships of the authors helped greatly in enabling the study to reach the number of journals and submissions that it reached. The analysis of the data was described well and carried out well, making it possible to replicate the experiment, in theory. As much of this study did depend on professional relationships, it's not clear if it could be replicated in practice, but given the nature of the study, this is expected. The authors are to be commended in particular for supplying the raw data supporting the analysis in a usable form as supplementary data, This improved my understanding of the work.

With regard to the 4 outcome measures (Compliance, Accuracy, Identifiability, and Utility) the scoring method is clear and well-described, but I felt like there were some important aspects of the data that were not addressed by the authors. With respect to compliance, it could be said that receiving 312 papers with 572 identifiers shows willingness to contribute, but the data in Table 2 suggest that reaching this level of compliance required a very hands-on approach by the editors. This suggests that compliance may be actually fairly hard to get. It's encouraging that 200 new antibody entries were created by authors, but it would have been good to see more data on the prevalence of other resources.

With regard to the accuracy goal, a user study of a group of authors going through the workflow would have a useful addition to the study to help determine where the trouble spots for authors arise. Are they in the lookup on the SciCrunch portal or, as suggested for the case of MGI, at the registry itself?

The identifiability pre-pilot results are consistent with the results we reached in our analysis of the Reproducibility Project: Cancer Biology set of papers. The authors assert that authors are able to perform the task accurately for software tools, but it is worth noting that software tools had low identifiability before and improved the least. Some discussion about why that is the case would be useful.

Regarding utility, evidence is presented that RRIDs are highly useful because an RRID can be entered into Google Scholar and a link to a paper mentioning that RRID can be retrieved. This is perhaps the weakest part of the study, because the utility criteria were that a query could be constructed to show all the publications in which the resource were used could be found. They report data that only 174 identifiers could be found in Google Scholar, but it's not clear how this data was obtained, not exactly what the numbers refer to. The exact query used for each search index should be supplied & the results themselves should have been stored. Given the lack of detail around these points, it's hard to judge if it's actually possible to conduct these queries in a systematic fashion. The discussion about the failure of researchers to include RRIDs in a consistent form shows the current method to get RRIDs inserted in the literature is not sufficient for widespread use, and the level of support required by the SciCrunch site maintainers (100 queries for 10000 searches) also suggests that this approach would not scale. Given the lack of an API for Google Scholar, the approach used is understandable, and it's noted that the RRID Resolver page at SciCrunch does link to a PubMed query for papers mentioning the resource, but there is a text mining API available for ScienceDirect papers which would have been a better choice for assessing presence in ScienceDirect. Overall, the study would have been improved by the addition of a data scientist & must be judged to have failed on the utility criteria as established. This doesn't mean RRIDs are shown not to be useful; on the contrary, it's clear that RRIDs do allow a researcher to find the actual resource used, so in that respect they're quite useful indeed and the resolver page is very helpful. There's a case for broader utility to be made, as well, with the example of the "Antibody data for this article" article enhancement on ScienceDirect.

It would also have been useful to see a more extended discussion of the sustainability of this project. For example, are journals to take this on as a means to add value, and if so, who pays for the maintenance of the SciCrunch resolver?

Overall, the authors have presented good quality data on the feasibility and usability of asking authors to contribute RRIDs to publications and the publishing community should consider this study when considering ways to enrich the literature for text and data mining purposes.

The takeaways for me are:

Just asking authors via the information for authors pages or via one-off emails does not work.

Letting authors enter RRIDs manually detracts from the utility of the identifiers. There's a big opportunity for machine learning approaches here to identify and suggest RRIDs at submission.

Non-open access articles inhibit research by preventing the full-text indexing of their articles by search indexes.

I have read this submission. I believe that I have an appropriate level of expertise to confirm that it is of an acceptable scientific standard.

# Peer review: Referee response for version 1

**DOI:** 10.5256/f1000research.7039.r8814

**Published:** 2015-06-05

**Authors:** Randi Vita

**Affiliations:** 1 Division of Vaccine Discovery, La Jolla Institute for Allergy and Immunology, La Jolla, CA, USA

This manuscript is overall well written with an appropriate title. The abstract provides an adequate summary and the design and explanation are sound. I believe the conclusions are justified and the data provided is adequate to support them. The subject is valuable to many researchers and provides an important service to researchers going forward. I have a few minor grammatical issues to report:

1. The sentence "The pilot project has focused on a limited number of resources - antibodies, software tools/databases, and model organisms" should be "The pilot project focused on a limited number of resources..."
2. in "accession number, as Genbank does for gene" should use "GenBank"
3. For sentence "Over the minimum 3-month window, each partner journal would request that authors supply RRIDs in a standard format as a citation to indicate the use of any of these three types of research resources."
  In this context, what the "three types of research resources" are is unclear.
4. The sentence "As these papers start to appear in PubMed Central where there is full text search, it should be possible to find papers for RRIDs through the National Library of Medicine." is a little confusing, maybe "As these papers start to appear in PubMed Central, where full text search is possible, papers utilizing RRIDs will be identifiable through the National Library of Medicine." is better? Yes, being  overly nit picky, but I had to read it twice to get it.
5. Verb tense should be reviewed throughout the manuscript to ensure consistency.
6. Uses of dashes such as "...from vendors - both solicited and unsolicited - and at.." occurs throughout the manuscript. This seems nonstandard?
7. In Figure 3, I could not see the pre-pilot bar. This could be due to something with my browser, but worth checking out.
8. In the Discussion, the sentence "The pilot RRID project has been highly successful in demonstrating the utility of a system to aid in identification of these three research resources in the literature" should reiterate what three research resources it is referring to.
9. Review the number of uses of the word "however" and how close to each other they occur. For example, "However, authors are encouraged in the citation format to include details about the particular instance of this antibody, namely, the vendor from which the antibody was purchased and the catalog, batch, and lot numbers. However, we..."

And lastly, the link referred to here "A list of the participating journals is available on the Force11 website ( https://www.force11.org/RII/SignUp)." did not provide a list of the multiple immunology journals in the Elsevier family that are participating. The page just shows the Elsevier logo.

I have read this submission. I believe that I have an appropriate level of expertise to confirm that it is of an acceptable scientific standard.
